# Tic Douloureux—Two Cases

**Published:** 1869-09-15

**Authors:** T. A. Smurr

**Affiliations:** Cleveland, Ohio


### CHICAGO MEDICAL JOURNAL

Vol. XXVII.—SEPTEMBER 1, 1869.—Nos. 17 and 18.

### COMMUNICATIONS.

### *Tic Douloureux—Two Cases. Their History, Pervantia and Results.*

BY T. A. SMURR, M.D., CLEVELAND, OHIO.

Case 1st. Mrs. A. B., age forty-two, fair complexion and cephalic temperament. Mother of one child, now grown up. Has not been enciente since birth of child, nor suffered from any irregularities of menses, nor any leucorrhœal discharge, but has enjoyed uniform good health. Nor has she been exposed to malaria. About six (6) months since, from some occult cause, a chain of morbid actions set in, resulting in octeritia alba, hyperæsthesia nervosa with hemorrhoids, and latterly great prolapsus ani, and vague and fugitive rheumatic pains, affecting especially the cervical muscles, and which the patient assured me were hereditary.

To use the patient's own words, she had tried every thing, and it did seem to her that doctors did not understand her case, or there was nothing in medicine which could do her good. Dispondency and want of faith might, therefore, be added to the complications. Appetite and sleep were both capricious, and, all things considered, the patient was a most unpromising one. She was placed upon the following:

| | | |
|---|---|---|
| PILL: ℞ | Sulph. quinia | gr. xxi; |
| | " Ferr. pulv. | ʒss; |
| | " Strych. | gr. ss; |
| | Sol. ext. Fel. Bovin | ʒi; |
| | M. Fiant Pill No. | xxi. |

S. One pill three times daily before eating.

Local treatment of both therapeutical and mechanical sort was employed for relief of the prolapsus. Under this system of treatment the patient rapidly improved, bowels became normal in action, tone was restored to the relaxed parts, the hyperæsthesia was subdued, and the cachexia improved. Sleep and appetite all that could be desired. She was now called from home, and was lost sight of for a time. On her return I was again sent for and found her suffering from a severe attack of tic douloureux of the portio dura nerve and its associate branches of the fifth pair of nerves. On inquiry found the attack had been anticipated by the re-developement of the cachexia, loss of appetite and insomnia, also rheumatic angina of the cervical muscles.

The bowels were constipated, and the renal secretions scant and high colored, dermal secretions almost *nil*. The teeth were all good but one molar, which was much discolored, and had been filled, and from which the gum—which otherwise seems healthy—was separated, and between which was secreted a small quantity of healthy pus. There was no sensitiveness on concussion against any of the teeth. A cathartic was ordered, to consist of pill hydrarg and extract colocynth, to be assisted after a time with an enema of oleaginous character, opiates were given to produce quiet and rest.

Called next day, found my patient no better. Bowels had been very thoroughly evacuated, but the tongue was still furred and the breath loaded. Kidneys acting more freely with less precipitate. Placed the patient upon the following:

| | |
|---|---|
| ℞ Sulph. quinia, | gr. xxvi; |
| " Strych., | gr. ss; |
| Pulv. sulph. Ferri, | ʒiss; |
| Aquæ cardamomi, | ℥vi; |
| Fl. ext. belladonna, | ℥ss; |
| Mixed. | |

S. A desert spoonful every four hours.

Called next day, Nov. 4, found patient was feeling easier and had begun to feel the action of both the quinia and belladonna. Lengthened the time of dose to six hours, continued treatment of same remedies, and ordered a generous diet of eggs, milk and beef tea, with ablutions of a solution of ammoni carbonas.

Nov. 5. Patient slept soundly during the night, was free from pain, but complained of the feeling in the ears and the effect of the light upon the eyes. Continued the treatment. Was called at 3 p. m., and found her suffering intensely and perfectly wild with pain and excitement. Now determined, notwithstanding the reputed antagonism of opium and belladonna, to use morphine hypodermically, which was done by throwing a half grain into the cellular tissue on inner side of humerus. In fifteen minutes the pain was very moderate, and in a half hour the pupils, which were largely dilated, begun to contract. At the expiration of an hour pupils were the size of a No. 6 shot, after which they seemed to have passed their maximum of contraction and began to dilate again. Injected a quarter of a grain more morphine. Withdrew the medicine she had been using, and substituted ℞ quinia, grs. v, every six hours, with opiates and Fowler's solution five drops, with Ferri chloride fifteen drops three times a day.

Nov. 6. Patient still suffered but not so acutely, was deaf and stupid from the effect of the quinia and morphia.

7th. Passed a pretty refreshing night, but represented the evening's exacerbation as having been very severe. Bowels moved of themselves. Breath fair and skin relaxed. Lessened dose but continued quinia. Was called at five and one-half p. m., and found her writhing with pain, which was again met by hypodermic medication, one-half grain morph. was followed in ten minutes by sleep.

9th. Patient in *statu quo*.

20th. Pain less intense, but still keeping its peculiar character.

11th. Much worse. A grain of morphine was used at two operations within the half hour. The pinnaæ of the ear and tip of the nose very tense with serous infiltration. The left eye was also closed by infiltration of the lids. Stomach irritable. Withdrew all medicines by the mouth, and ordered an enema of oil and turpentine. Beef tea, chicken broth, with a slight flavoring of dilute hydrocyanic acid, carefully manipulated, soon settled the stomach. Opiates used hypodermically.

12th. Patient still suffering when not under the influence of opiates. Again urged the extraction of the tooth referred to before as the probable offending cause, but was put off for the time. Ordered a lotion of chloroform and Tr. Rad. aconite *aa* to be applied to painful parts. P. M., as patient seemed to be very weak, ordered milk punch, with specific directions as to its preparation and exhitition. sul. morph. one-half grain, and left morph. and aconite during night.

13th. Found patient suffering but little pain, but very restless. Slept none during the night. Pulse 124 to 130. Bowels had moved, skin was moist, secretions from kindeys dark and small in quantity. Was at a loss to account for the feeble, rapid pulse. She complained bitterly of the punch, and upon inquiry found that our Emerald friend, true to the record of her race, had blundered, and had given an entire pint of the very best rye whisky during the previous *ten hours*, and had only desisted because the 'supply had failt enteerely, and the doctor, God bless him, wud ba so disapinted," and so he was. But despite all she was easier, nor was she in the least intoxicated from the spirits, which was most remarkable, not being habituated to the use of any form of liquors.

14th. Still feeling comfortable. P. M. Her catamenia appeared—normal period.

From this to 17th but little change. Evening and morning paroxysms still continued, and the menstrual discharge is light colored and less than usual. P. M. The menses has stopped gradually—usually continued four to six days.

18th. Paroxysms required more morph. to control them than usual. Patient not so well.

19th. Examined mouth, tongue foul, but do not see that my suspicions of the noxious character of tooth were well founded, as it seems not at all tender or swollen. Thus rested the patient until about the 26th, in a state of semi-comfort, sometimes better and again the reverse. In the evening of that day gum along the left side began to swell, and on the 28th made an incision of about one inch in extent, giving exit to a large accumulation of healthy pus. Exploration revealed no caries of alveolus or teeth. I now confidently looked for a respite, if not a recovery. But the 29th came, and with it the same roll of pain, and but little change of medicament. Then the 30th dawned full of pain, and almost despairingly I turned the case over again, hoping some light as to cause or treatment might be poured upon a most unpromising subject. Bowels had not moved for a few days, and as breath was again becoming fetid and stomach irritable, the patient was ordered ℞ Pill, Olem Tiglii., containing one drop each of fresh oil. S. To be taken at once and followed by one every two hours until bowels were freely moved. ℞. Vinum colchic. sem. was ordered every two hours with opium sufficient to control pain.

Dec. 1st. Bowels had moved very freely, and much scybala of a light clay colored interior, but exteriorly of a sanious slate color, and very offensive. Patient had passed a very refreshing night and felt very hopeful. The vinum colchic. was now combined with pepsin wine, and a very generous animal diet, and vigorous shampooing of all the muscles was directed. The patient improved daily.

On the 10th the bowels became again sluggish, and in a day or two after a relapse was seriously apprehended, but a brisk action from Oleum Tiglii and all pain was at an end.

The patient made a slow but continuing recovery, which has been uninterrupted by return of neuralgic pain to present writing, some nine months, and further, retains the condemned tooth, which, since the thorough incision referred to, has not given the least trouble.

